# 3 Lobes of Extracardiac Hemangioma

**DOI:** 10.1016/j.jaccas.2024.102406

**Published:** 2024-08-07

**Authors:** Atsuyuki Mitsuishi, Yujiro Miura, Shingo Hosogi, Miho Tsutsui, Hiroaki Kitaoka

**Affiliations:** a Department of Cardiovascular Surgery, Kochi Medical School Hospital, Kochi, Japan; b Department of Cardiology, Hosogi Hospital, Kochi, Japan; c Department of Pathology, Kochi Medical School Hospital, Kochi, Japan; d Department of Cardiology and Geriatrics, Kochi Medical School Hospital, Kochi, Japan

**Keywords:** extracardiac, hemangioma, tumor, 3 lobes

## Abstract

Cardiac hemangioma is a rare benign disease, and extracardiac tumors consisting of 3 lobes are exceptionally rare. Preoperative diagnosis is extremely difficult, and it is important to evaluate the disease through a multifaceted examination.

## Graphical Abstract

**Figure:**
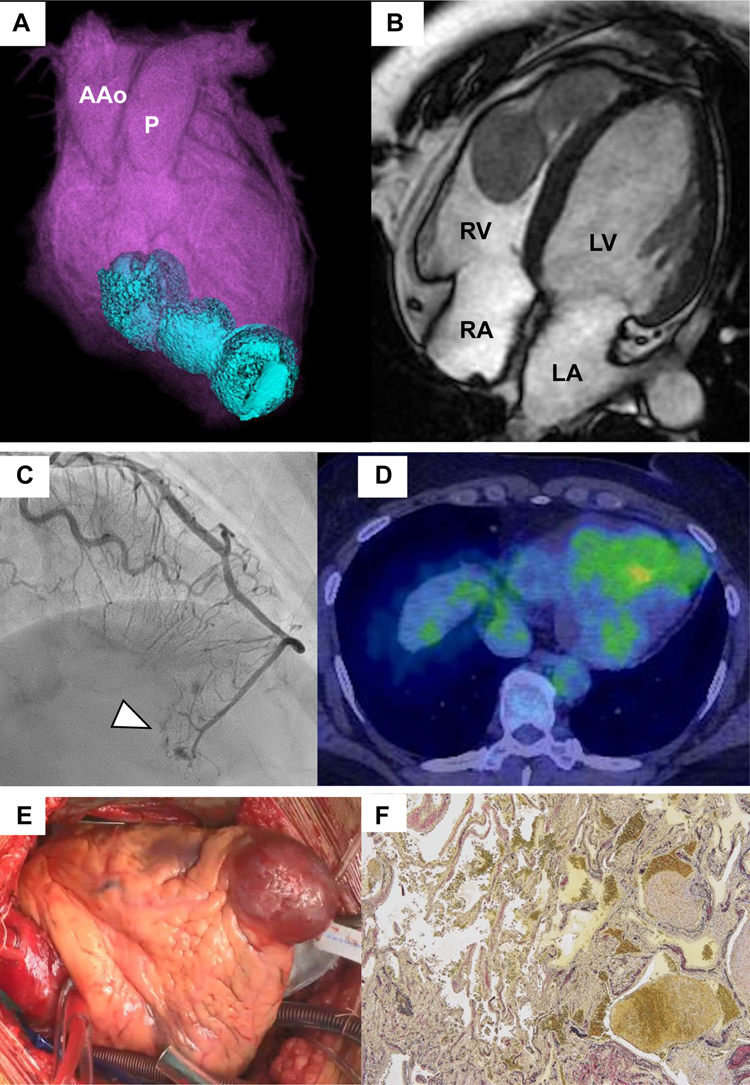

A 53-year-old woman with a history of asthma and hypothyroidism underwent preoperative examination at another hospital for a benign right mammary tumor, and echocardiography revealed abnormal structures in the right ventricle. Right ventricular (RV) myxoma and hemangioma were suspected on the basis of cardiac computed tomography (CT) (Figure 1A) and cardiac magnetic resonance (CMR) (Figure 1B, Video 1), and she was referred to the cardiology and cardiovascular surgery departments of our hospital (Kochi medical school hospital, Kochi, Japan). Coronary angiography (CAG) revealed a feeding artery from the left descending coronary artery (Figure 1C, Video 2), and a preoperative biopsy was performed simultaneously because of suspicion of a malignant cardiac tumor that did not seem to have received curative surgical treatment. Histopathologic confirmation was believed to be a necessary option other than the treatment approach; however, tumor cells could not be collected. Positron emission tomography (PET) combined with CT revealed a concentration of a maximum standardized uptake value of 5.0 in the mass predominantly located in the right ventricle (Figure 1D). During the surgical procedure, we observed a tumor extending beyond the heart’s surface. We performed right ventriculostomy into the apex of the heart along the acute marginal artery. Despite partial adhesion of the myocardium and tumor, it was possible to dissect clearly and detach the boundary (Figure 1E, Video 3). The rapid pathology results established a diagnosis of benign hemangioma, resulting in a complete resection of the tumor. We used a felt strip and performed double layers of RV suture closure.

**Figure 1:**
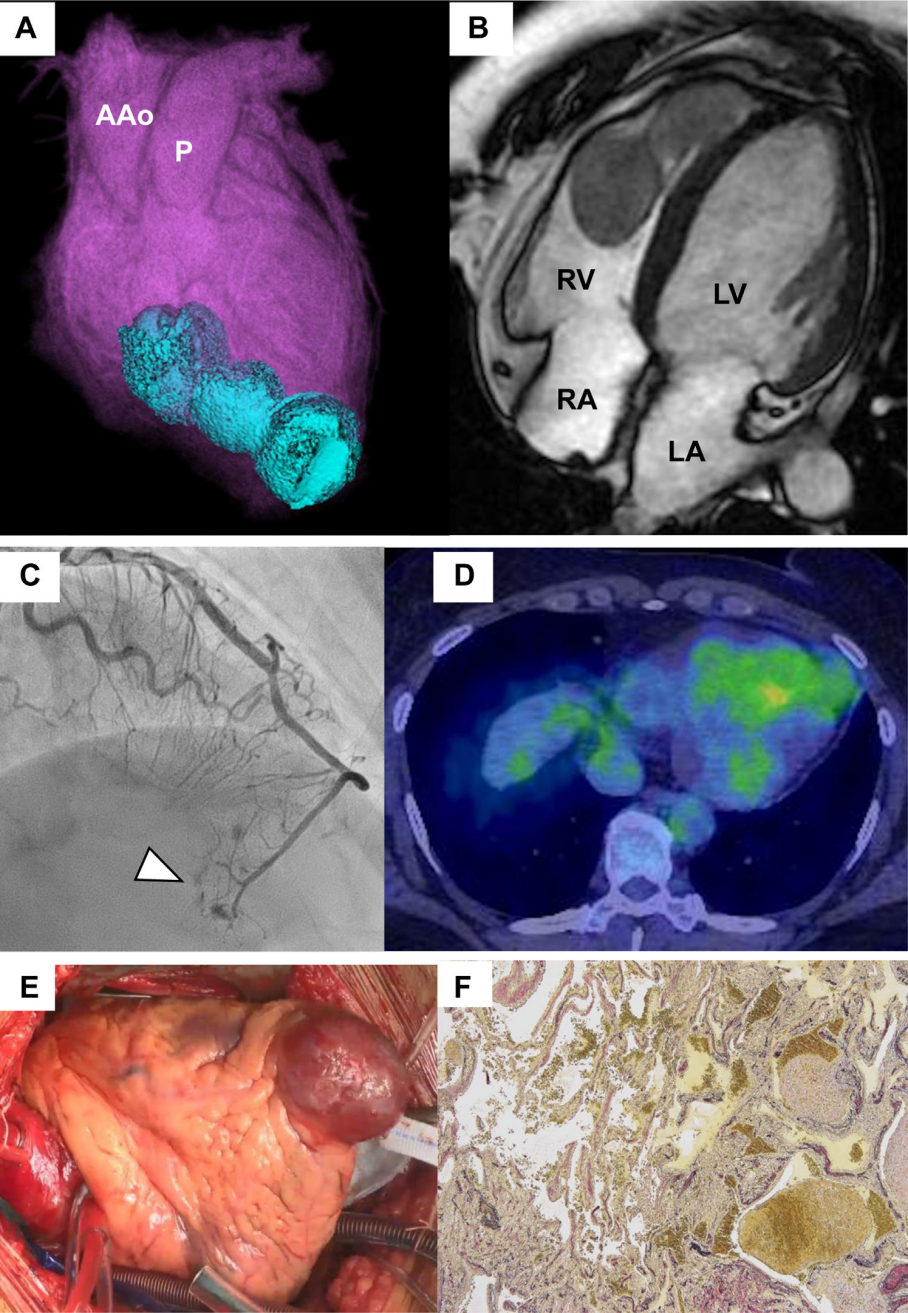
Multimodality Imaging of Cardiac Hemangioma (A) Three-dimensional cardiac computed tomography. revealed an extracardiac hemangioma with 3 lobes (in blue). (B) Magnetic resonance imaging showed that the hemangioma was T2 high and T1 low, and the surface had smooth and regular borders. (C) Coronary angiography revealed a feeding artery from the left descending coronary artery to the hemangioma (arrowhead). (D) Positron emission tomography revealed a maximum accumulation of a standardized uptake value of 5.0 in the right ventricle (RV). (E) Operative view of the extracardiac hemangioma at the right ventricular apex. (F) Elastica van Gieson stain revealed elastic fibers in the vascular-like structures of the tumor, which was considered a hemangioma. AAo = ascending aorta; LA = left atrium; LV = left ventricle; P = pulmonary artery; RA = right atrium.

On the postoperative pathology examination, the 3 lobes of the hemangioma measured 22 × 17 × 8 mm, 30 × 15 × 12 mm, and 45 × 20 × 20 mm from the outside. We observed dense, vascular-like structures with irregular anastomoses and dilatations, scattered with old and new thrombi. The cells lining the vascular-like structures were small and without significant atypia, and Elastica van Gieson staining revealed elastic fibers in the vascular-like structures (Figure 1F). We classified this tumor as a hemangioma, specifically a cavernous hemangioma. There were no obvious signs of malignancy.

## Discussion

Cardiac hemangiomas are extremely rare,^1^ and a 3-lobed extracardiac hemangioma is exceptionally rare. Given the risk of life-threatening complications, surgical treatment is a reasonable option for all patients with cardiac hemangiomas.^1^

Cardiac tumors can affect the endocardium, myocardium, or epicardium. The cavernous and intramuscular types of tumors tend to be infiltrative, whereas the capillary type tends to be circumscribed.^2^ In our case, the hemangioma included the myocardial tissue; however, the primary site could not be determined. Only one-third of these patients have received an accurate diagnosis of hemangioma before surgery.^3^ In our case, CAG revealed a feeding artery to tumors, and PET combined with CT did not show a high accumulation of tumors, which appeared benign. Moreover, on the basis of cardiac CT and CMR, which showed smooth tumor morphology with regular borders, T2 high and T1 low findings on CMR, and positive contrast findings on CT, the histologic differentiation was myxoma or hemangioma, which had a relatively benign nature. However, because the tumor protruded into the tissue outside the apex, a definitive diagnosis could not be made before the pathologic findings were examined.

## Funding Support and Author Disclosures

The authors have reported that they have no relationships relevant to the contents of this paper to disclose.

## Appendix

Video 1 Cardiac Magnetic Resonance Imaging showed that the hemangioma was T2 high and T1 low, and the surface had smooth and regular borders.

Video 2 Coronary Angiography Imaging revealed a feeding artery from the left descending coronary artery to the hemangioma.

Video 3 Intracardiac view with video-assisted cardioscopy showing that the hemangioma was penile and easily movable.
